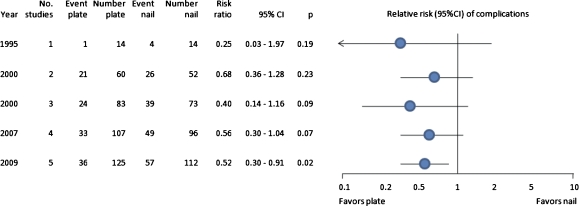# Treatment of humeral shaft fractures–meta-analysis reupdated

**DOI:** 10.3109/17453674.2010.504611

**Published:** 2010-07-16

**Authors:** David J. Heineman, Mo Bhandari, Sean E. Nork, Kees-Jan Ponsen, Rudolf W. Poolman

*Sir*—In April 2010 a meta-analysis with the title “Plate fixation or intramedullary fixation of humeral shaft fractures” was published by our group in Acta Orthopaedica 2010; 81 (2): 216-23. In this article we stated that there was no statistically significant difference in primary and secondary outcomes between plates and intramedullary nails in humeral shaft fractures.

Just after our meta-analysis was accepted for publication in Acta Orthopaedica, Putti et al. (2009) published a new randomized controlled trial comparing nails to plates in humeral shaft fractures making our findings obsolete.

To illustrate the need for regular updates in meta-analysis we conducted a cumulative meta-analysis now including Putti and co-workers study. Putti et al. included 34 patients, after randomization 16 patients were in the nailing group, 18 patients were assigned to the plating group. It is interesting to see that our primary outcome is different now and favors plates over nails (RR 0.52, CI 0.30–0.91, p = 0.02, see the Figure). Therefore we would like to state that the risk of a complication is lower when plating a fracture of the humeral shaft than when using an intramedullary nail.

In our primary meta-analysis we stated that there were some limitations to our study: first of all the small number of patients that could be included. With this new study only 34 patients were added, giving a total of 237 patients. The problem of heterogeneity remains as well with this extra study added.

Still, our cumulative meta-analysis shows the strengths of meta-analysis in providing clinicians up-to-date best evidence and help them in clinical decision making. However, to find the definitive answer on optimal treatment strategies for humeral shaft fractures a large scale RCTs is needed reporting on patient important endpoints such as complication rate and validated patient reported outcomes.

**Figure:**